# The accuracy of an extramedullary femoral cutting system in total knee arthroplasty in patients with severe coronal femoral bowing: a radiographic study

**DOI:** 10.1186/s13018-022-03140-2

**Published:** 2022-05-07

**Authors:** Qianjin Wang, Xiaofeng Zhang, Tianshu Shi, Zhengyuan Bao, Bin Wang, Yao Yao, Dengxian Wu, Zheng Liu, Honggang Cai, Dongyang Chen, Jin Dai, Qing Jiang, Zhihong Xu

**Affiliations:** 1 State Key Laboratory of Pharmaceutical Biotechnology, Division of Sports Medicine and Adult Reconstructive Surgery, Department of Orthopedic Surgery, Nanjing Drum Tower Hospital, The Affiliated Hospital of Nanjing University Medical School, 321 Zhongshan Road, Nanjing, 210008 Jiangsu People’s Republic of China; 2 Branch of National Clinical Research Center for Orthopedics, Sports Medicine and Rehabilitation, Beijing, People’s Republic of China; 3 Division of Sports Medicine and Adult Reconstructive Surgery, Department of Orthopedic Surgery, Nanjing Drum Tower Hospital Clinical College of Nanjing Medical University, 321 Zhongshan Road, Nanjing, 210008 Jiangsu People’s Republic of China

**Keywords:** Total knee arthroplasty (TKA), Femoral bowing, Extramedullary alignment, Intramedullary alignment

## Abstract

**Background:**

Intramedullary (IM) femoral alignment instrument is imprecise for the coronal alignment in total knee arthroplasty (TKA) in patients with severe lateral bowing of the femur, while the extramedullary (EM) alignment system does not depend on the structure of the femoral medullary cavity. The aim of this retrospective study was to compare the accuracy of postoperative limb alignment with the two femoral alignment techniques for patients with severe coronal femoral bowing.

**Methods:**

From January 2017 to December 2019, patients with end-stage knee osteoarthritis and coronal femoral bowing angle (cFBA) ≥ 5° who underwent total knee arthroplasty TKA at our institution were enrolled in the study. The postoperative hip-knee-ankle (HKA) alignment, femoral and tibial component alignment between the IM group and the EM group were compared on 5° ≤ cFBA < 10° and cFBA ≥ 10°.

**Results:**

In patients with 5° ≤ cFBA < 10°, no significant differences were observed in the EM group and IM group, including preoperative and postoperative parameters. However, when analyzing the patients with cFBA ≥ 10°, we found a significant difference in postoperative HKA (4.51° in the IM group vs. 2.23°in the EM group, *p* < 0.001), femoral component alignment angle (86.84° in the IM group vs. 88.46° in the EM group, *p* = 0.001) and tibial component alignment angle (88.69° in the IM group vs. 89.81° in the EM group, *p* = 0.003) between the two groups. Compared to the EM group, the IM group presents a higher rate of outliers for the postoperative HKA and femoral components.

**Conclusions:**

The study showed that severe lateral bowing of the femur has an important influence on the postoperative alignment with the IM femoral cutting system. In this case, the application of EM cutting system in TKA will perform accurate distal femoral resection and optimize the alignment of lower limb and the femoral component.

## Introduction

There are many factors that affect the outcome of total knee arthroplasty (TKA) [1, 2], the prosthetic placement and overall limb alignment has been demonstrated to be most influential in determining implant survival [3, 4]. Although some research suggests that coronal alignment after TKA has no significant effect on postoperative knee joint function and prosthesis life [5], the accuracy of the femur resection and the alignment restoration of the lower limb still play an important role in the longevity of prosthesis and the joint function. The precise of femur and tibia resection is the key for restoring a neutral alignment within 3° varus or valgus to the mechanical axis [6, 7]. Currently, tibia resection mainly uses extramedullary cutting guides, while femur resection has several choices such as conventional intramedullary (IM) guides, extramedullary (EM) guides, computer-assisted navigation surgery and patient-specific instruments. Previous studies have shown that IM is more accurate than EM guides [8, 9]. With the development of EM devices, recent studies show that there is no significant difference in accuracy between the two methods [10–12]. Due to excessive dependence on the structure of the femoral medullary cavity, the IM techniques cannot guarantee the accuracy of femur resection in patients with extra-articular deformities, femoral medullary cavity lesions or femoral shaft curvature deformities. Unlike Western populations, femoral lateral bowing is more common in Asians [7, 13, 14]. Studies have shown that the femoral lateral bowing may affect the alignment of the lower limbs with the IM technique [15, 16]. Many studies have reported that the accuracy of the computer-assisted TKA is higher than that of the IM [17–19], particularly in cases of femoral extra-articular deformity [20]. But in fact, the computer-assisted technology has its inherent shortcomings, such as higher cost, longer operation time, and a longer learning curve for surgeons. However, there was no previous studies on the accuracy of extramedullary systems in TKAs with severe coronal femoral bowing. Our team have designed and reported an extramedullary femur cutting system (FEM-X1; designed by ZHX, Kerunxi, Jiangsu, CHN), which have shown accurate alignment and desired clinical outcomes in clinical applications [21]. The present study is to investigate whether the new EM alignment technique is more conducive to restoring the alignment for patients with excessive coronal femoral bowing than IM.

## Patients and methods

### Patients

**Fig. 1:**
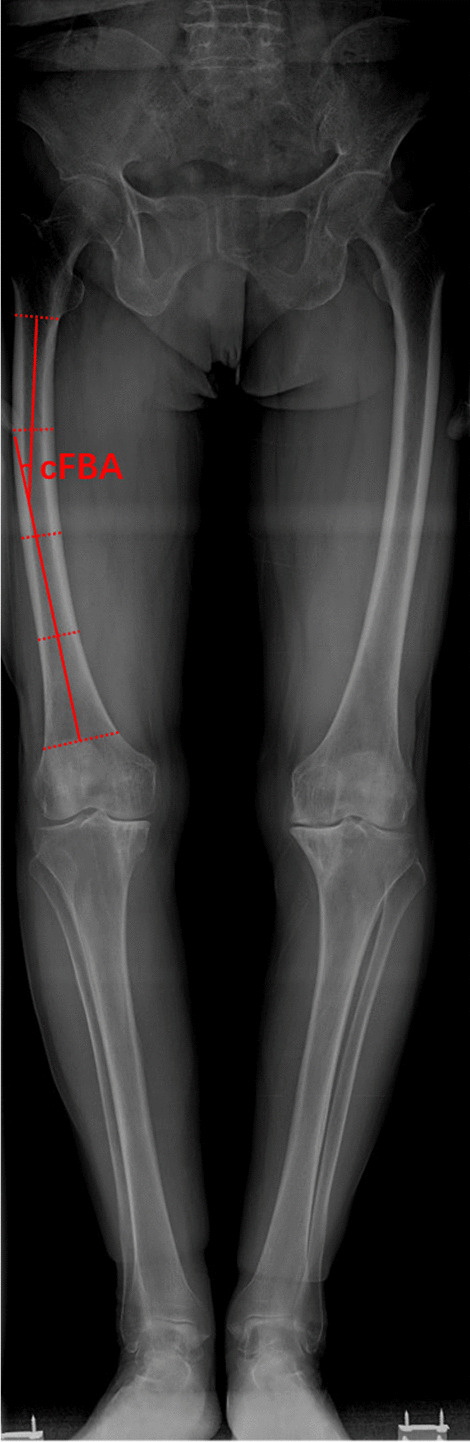
Measurement of femoral lateral bowing angle. cFBA (coronal femoral bowing angle): the angulation between the line that best described the midpoint of the endosteal canal in proximal and distal quarters, respectively

From January 2017 to December 2019, consecutive patients with end-stage osteoarthritis received primary TKA in our department were retrospectively analyzed. According the study by Yau et al. [13], we divided the femoral diaphysis into four equal parts on full-limb anteroposterior radiograph and the coronal femoral bowing angle (cFBA) of the femur was defined as the angulation between the line that best described the midpoint of the endosteal canal in proximal and distal quarters, respectively (Fig. 1). There was no preference for surgeons in femoral resection technique selection for patients. Patients with cFBA ≥ 5° defined as severe femoral lateral bowing were enrolled in this study and any of the following reasons were excluded: (a) valgus deformity of the affected limb (10 knees); (b) previous surgery of the limbs or the pelvis (1 knee); (c) incomplete radiographic records (4 knees); (d) non-standard standing when taking radiology exams (both patella facing the front) (14 knees); (e) revision prosthesis was used for initial primary TKA (2 knees). Finally, 181 patients met the criteria were included in this study. Gender, age, body mass index (BMI), preoperative and postoperative full-limb anteroposterior radiographs were extracted from clinical records for analysis. The demographic characteristics are expressed in Table 1. This study has been approved by the Ethics Committee of Nanjing Drum Tower Hospital (No. 2016-016) and obtained the consent of patients.

**Table 1:** The summary of patients’ demographic and preoperative data

	IM (110)	EM (71)	*p* Value
Age (years)	69.89 ± 7.29	70.80 ± 7.69	0.422
Sex (female/male)	102/8	59/12	0.224
BMI (kg/m^2^)	24.63 ± 3.93	25.26 ± 4.32	0.313

The values are expressed as mean ± SD or numbers of patients

*IM* intramedullary guide group, *EM* extramedullary guide group, *BMI* body mass index

*p* < 0.05 was considered to indicate statistical significance

### Preoperative preparations

**Fig. 2:**
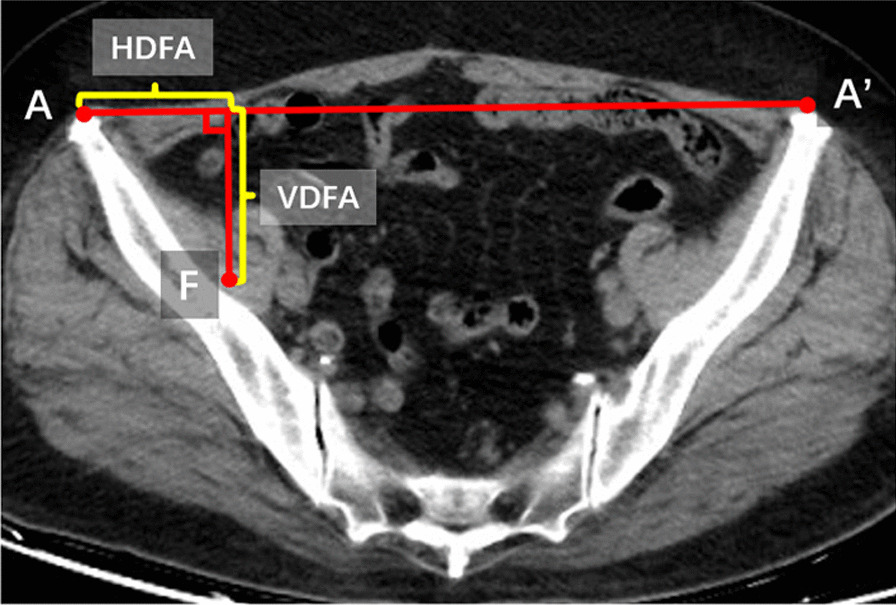
Relevant parameters were measured on CT radiograph before operation with the extramedullary femoral cutting system. A/A′: Anterior superior iliac spine. F: The femoral head center

In the IM group, distal femoral cutting block was placed based on the femoral valgus angle measured preoperatively on the standing full-length anteroposterior radiographs. In the EM group, computer tomography (CT) scan of the pelvis paralleled with bilateral anterior superior iliac spine was taken for surgical planning. The femoral head center of the affected limb was chosen first, then slide the mouse wheel to the version of bilateral anterior superior iliac spine. The distance of bilateral anterior superior iliac spine (A–A′) and the distance of bilateral femoral head center (F–F′) were measured. We defined the distance from the projection point of the center of the femoral head on A–A′ to the apex of the anterior superior iliac spine on the affected side is recorded as HDFA and the vertical distance from the center of the involved femoral head to A–A′ as VDFA. The parameters of EM system were referred to the value of HDFA and VDFA (Fig. 2).

### Surgical technique

**Fig. 3:**
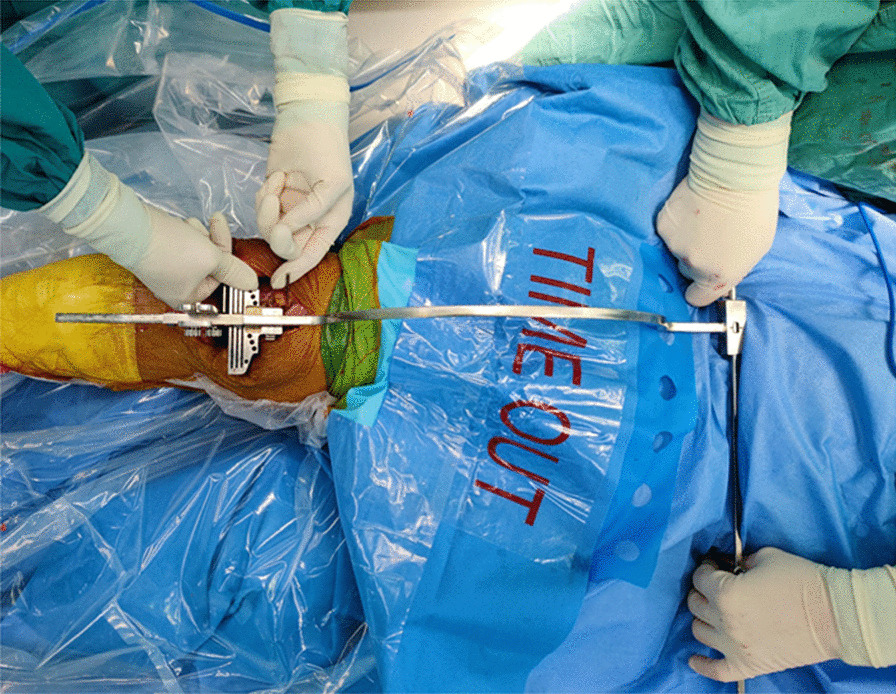
TKA with EM alignment guide

An anterior longitudinal incision with a medial para-patellar approach was used in all TKAs. After full exposure to the surgical field, the tibial resection was performed to be 90° to the mechanical axis of tibia on the coronal plane and 3° posterior slope on the sagittal plane with an extramedullary instrument. The resection of femur was performed from the distal femur to the anterior and the posterior femur, and except for the distal femoral resection, all procedures were consistent between the two surgical procedures. When resecting the distal femur in the IM group, an alignment rod was inserted into femoral medullary cavity, and a distal femoral cutting block was connected according the femoral valgus angle measured preoperatively. In the EM group, the distal femur resection was performed using the extramedullary instruments which consists of a horizontal bar marked with scales and an arc-shaped bar vertical to it. There are two pegs at both ends of the straight rod, which are fixed to the apex of the anterior superior iliac spine during the operation. The cutting jig is equipped in the end of the arc bar, sticking on a nail marking the center of the knee joint. During surgery, the surgeon marks the intersection of transepicondylar line and Whiteside line as the center of distal femur, then fixes a nail in this point before installing the EM femoral resection instruments. Then, the operated leg was stretched and flatted on the operating table. After adjusting the EM equipment, the surgeon fixes the two pegs of the extramedullary guides symmetrically on the anterior superior iliac spine, while an assistant gently supports the end of the mechanical bar to adapt the orientation of the rod controlled by the surgeon, then another assistant moves the femur so that the center of the knee joint is directly below the slot of the guide, then the assistant pulls the guide’ slot into the knee joint and fixes the resection block (Fig. 3). During surgery with EM guide, the sagittal mechanical alignment was controlled by the distal block, which was described in detail in our previous research [21]. Apart from distal femur resection, the basic surgical instruments were the same in both groups and all femoral components were placed with a 3°–5° external rotation toward the transepicondylar line.

### Radiographic measurements

**Fig. 4:**
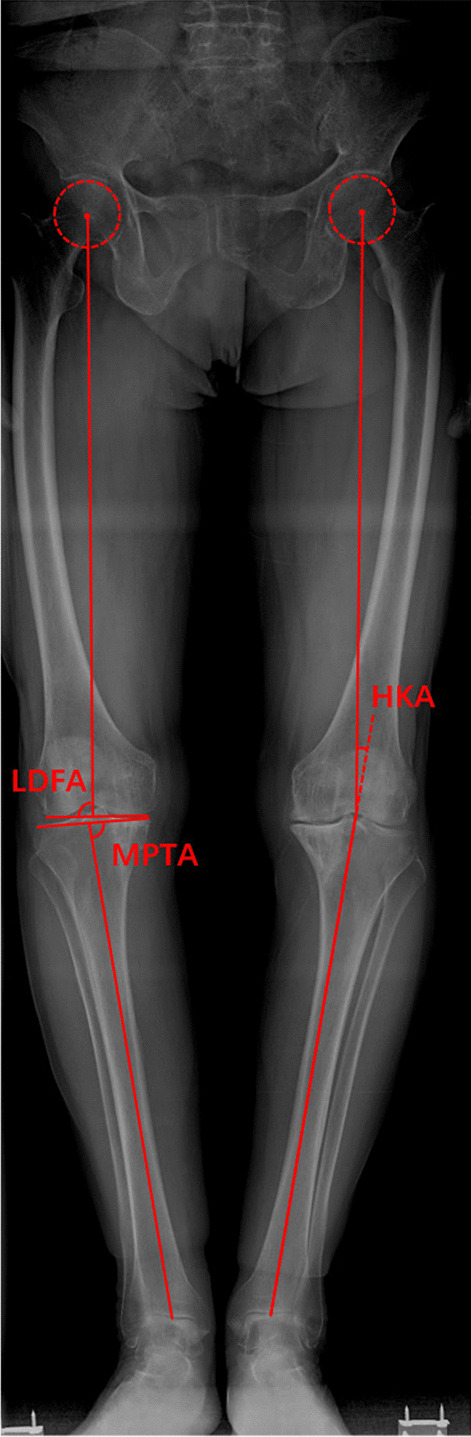
Measurement of preoperative radiological parameters

**Fig. 5:**
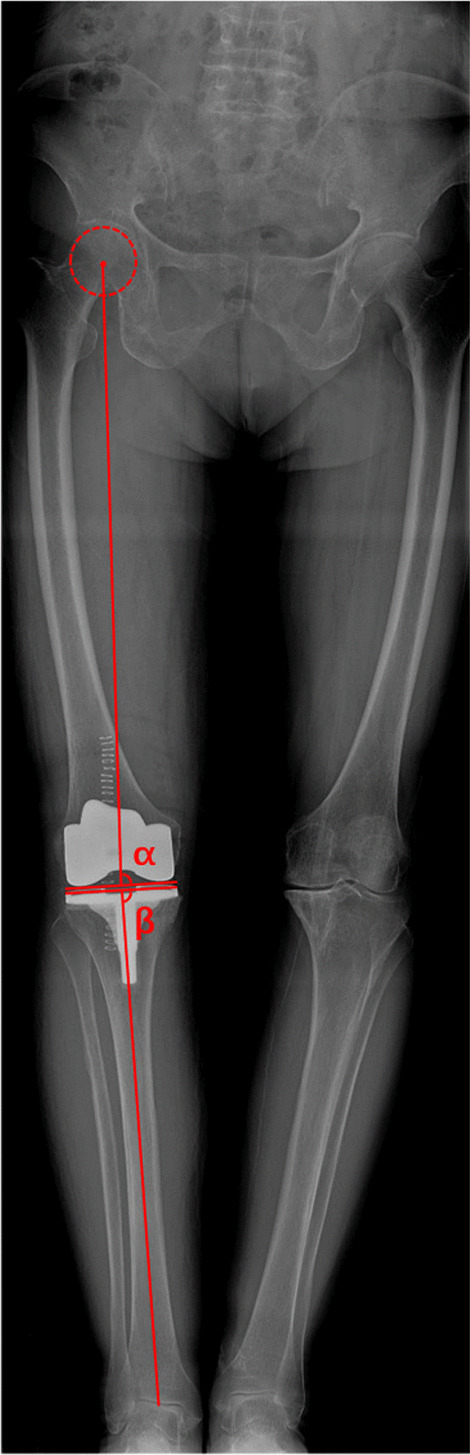
Measurement of postoperative radiological parameters

All radiological parameters were measured using a scanner viewer (RadiAnt DICOM Viewer 2020.1). The minimum value of the measured parameter is 0.1. Standing full-length anteroposterior radiographs were obtained with standard radiographic technique [22] from all patients before and after operation. When taking radiographs, patients were required to stand with both legs fully extended, with feet slightly internal rotated and bilateral patella facing forward parallel when radiograph taken. Except cFBA, other preoperative imaging angles including the hip-knee angle (HKA) of the involved limb, the distal femoral lateral angle (LDFA), the proximal medial angle of the tibia (MPTA) were measured (Fig. 4), the angles related to the position of the prosthesis after the operation including the HKA angle, the femoral component angle (α) and the tibial component angle (β) were analyzed as well (Fig. 5). The outlier was defined as the HKA outside the range of 0 ± 3° and the femoral component alignment angle outside the range of 90 ± 3°. All the data were measured by two orthopedic surgeons (QW, DW) who were not involved in the surgery.

Lateral distal femoral angle (LDFA): the lateral angle between the mechanical axis of the femur and the line tangent to the most distal point of medial and lateral condyles of the femur; medial proximal tibial angle (MPTA): the medial angle between the mechanical axis of the tibia and the proximal tibial joint line; hip-knee-ankle angle (HKA): the lateral angle between the mechanical axes of the femur and tibia.

Femoral component alignment angle (α): the medial angle between the mechanical axis of the femur and the distal femoral component line; tibial component alignment angle (β): the medial angle between the mechanical axis of the tibia and the proximal tibial component line.

### Statistical analysis

IBM SPSS statistics 26.0 software was used for statistical analysis. T test was used to analyze the data of hip, knee, ankle, and tibial prosthetic valgus angle, and the person Chi-square test was used to analyze the count data such as age, gender and the outlier of postoperative alignment. The difference is statistically significant when *p* < 0.05.

## Results

A total of 181 knees with excessive femoral bowing (cFBA ≥ 5°) met eligible criteria for analysis (120 knees with cFBA ≥ 5° and < 10°, 61 knees with cFBA ≥ 10°). In the patients with cFBA ≥ 5° and < 10°, no significant difference was observed in the EM group and the IM group, including preoperative and postoperative parameters (Table 2). However, when analyzing the patients with cFBA ≥ 10°, we found a significant difference in postoperative HKA (4.60° ± 2.33 in the IM group vs. 2.18° ± 2.03 in the EM group (*p* < 0.001)), α (86.84° ± 1.53 in the IM group vs. 88.46° ± 2.23 in the EM group (*p* < 0.001)) and β (88.69° ± 1.35 in the IM group vs. 89.81° ± 1.37 in the EM group (*p* < 0.001)) between the two groups (Table 3). In patients with cFBA ≥ 10°, 20 (55.6%) knees with postoperative HKA deviated from the neutral axis over ± 3°in the IM group, compared with 8 (32.0%) in the EM group, 17 (47.2%) femoral component alignment deviated from the neutral axis over ± 3° in the EM group, compared with 6 (24.0%) in the IM group. The outlier rate between the two groups is shown in Table 4.

**Table 2:** The summary of alignment in patients with cFBA ≥ 5° and < 10°

Variable	IM (74)	EM (46)	*p* Value
Preoperative HKA	10.37 ± 5.46	11.51 ± 4.77	0.248
LDFA	90.70 ± 2.87	90.09 ± 2.23	0.227
MPTA	83.56 ± 8.95	83.74 ± 3.52	0.895
Postoperative HKA	2.81 ± 2.12	2.56 ± 1.90	0.520
Femoral component alignment (α)	88.41 ± 2.04	88.16 ± 1.90	0.505
Tibial component alignment (β)	88.91 ± 1.26	89.30 ± 1.20	0.091

The values are expressed as mean ± SD

*IM* intramedullary guide group, *EM* extramedullary guide group, *LDFA* lateral distal femoral angle, *MPTA* medial proximal tibial angle

*p* < 0.05 was considered to indicate statistical significance

**Table 3:** The summary of alignment in patients with cFBA ≥ 10°

Variable	IM (36)	EM (25)	*p* Value
Preoperative HKA	11.90 ± 6.48	14.27 ± 6.00	0.153
LDFA	88.58 ± 3.32	87.48 ± 4.44	0.271
MPTA	84.56 ± 2.87	83.16 ± 2.88	0.067
Postoperative HKA	4.51 ± 2.35	2.23 ± 2.05	< 0.001
Femoral component alignment (α)	86.84 ± 1.53	88.46 ± 2.23	0.001
Tibial component alignment (β)	88.69 ± 1.35	89.81 ± 1.37	0.003

The values are expressed as mean ± SD

*IM* intramedullary guide group, *EM* extramedullary guide group, *LDFA* lateral distal femoral angle, *MPTA* medial proximal tibial angle, *HKA* hip-knee-ankle angle

*p* < 0.05 was considered to indicate statistical significance

**Table 4:** The outlier rate between the two groups

cFBA	Variable	Outlier (%)		*p* Value
		IM	EM	
≥ 5°and < 10°	Postoperative HKA	25 (33.8)	13 (28.3)	0.527
	Femoral component alignment (α)	19 (25.7)	11 (23.9)	0.828
	Tibial component alignment (β)	3 (4.1)	2 (4.3)	0.938
≥ 10°	Postoperative HKA	20 (55.6)	8 (32.0)	0.069
	Femoral component alignment (α)	17 (47.2)	6 (24.0)	0.066
	Tibial component alignment (β)	2 (5.6)	1 (4.0)	1.000

The values are expressed as patients (n (%))

*cFBA* coronal femoral bowing angle, *IM* intramedullary guide group, *EM* extramedullary guide group, *LDFA* lateral distal femoral angle, *MPTA* medial proximal tibial angle, *HKA* hip-knee-ankle angle

*p* < 0.05 was considered to indicate statistical significance

## Discussion

The most important finding of this study is that the use of a new EM femoral resection system may obtain a more accurate placement of femoral components and reconstructed lower extremity alignment than conventional IM system when performing TKA on patients with excessive coronal femoral bowing deformity.

The outcome of TKA is dependent on the orientation of femoral and tibial component and reconstructed lower extremity alignment that is within 3° of neutral [1, 23–26]. Accurate distal femoral bone resection perpendicular to the mechanical axis of femur is critical for achieving ideal reconstructed mechanical axis, prolonging the longitivity of prosthesis and improving satisfaction of patients [13]. Unfortunately, there are still many TKAs failing to achieve the ideal alignment with conventional guides, and the morphology of femoral shaft accounted. The conventional TKAs with intramedullary femoral resection system refer to as the anatomical axis of the femur. In order to cut the femur perpendicular to the MA, the resection block of distal femoral is adjusted according to the valgus correction angle of the distal femur. Many surgeons routinely use 5–6° valgus correction angle for distal femoral resection [27]. However, the valgus correction angle is influenced by femoral bowing [16, 28, 29]. Coronal bowing of the lower extremity is common among Asians with advanced osteoarthritis of the knee, a study of patients with end-stage primary osteoarthritis showed a high prevalence of coronal femoral bowing in Chinese populations [30]. Many studies have reported the influence of femoral lateral bowing on the position of component and postoperative TKA alignment [31–33]. Recent studies dealing with femoral anatomical structure found that the femoral valgus angle has great variations in individuals, which indicates that an arbitrarily fixed angle for distal femoral resection may lead to unacceptable planning errors; this is likely one of the main reasons for such errors [14, 34]. Moreover, standing full-length anteroposterior radiographs is not widely performed in clinical institutions, many patients with excessive femoral lateral bowing are overlooked in preoperative planning with short-film radiographs of the knee. In addition, the accuracy of radiographic measurement is inevitably affected by the lower limb rotation and the factors concerned with equipment [35–37]. Thus, the planned valgus correction angle of intramedullary system cannot be hold true.

Many studies have reported navigation or computer-assisted TKA to improve the accuracy of reconstructed lower extremity alignment [38], however, the high cost and practical complicities impede the widespread application of these techniques. In recent years, with the development of modern surgical technologies, several EM alignment guides have been invented and verified effective. Relative research works have reported that many novel EM systems were comparable with the IM technique on coronal alignment. Matsumoto et al. [10] located the femoral head with an image intensifier and have reported that there is no significant difference in postoperative alignment between IM and EM.

In our study, we developed a method for locating the femoral head center precisely on the computed tomography image of the hip (Bilateral anterior superior iliac spine included). Previously, we have initially confirmed the practicability and accuracy of the EM system and the satisfaction of early clinical outcomes [21]. In this research, radiological results showed no significant difference between the IM group and the EM group in patients with femoral lateral bowing < 10°, which further indicates that the accuracy of the EM system for performing the distal femoral bone resection. While in subjects with femoral bowing ≥ 10°, the EM group has a better LDFA and HKA postoperatively than IM group. During the IM operation, the surgeon adjusted the distal femoral valgus osteotomy based on the femoral valgus angle preoperatively measured on weight bearing whole leg radiographs. The inaccuracy caused by femoral bowing is that the entry point and direction of the intramedullary guide are difficult to control, especially in patients with excessive femoral bowing [39]. Extramedullary systems use the femoral center and the knee center to precisely locate the mechanical axis. In this way, surgeons can restore femoral alignment without relying on the structure of the femoral medullary cavity.

This study has several limitations. First, this was a retrospective study mainly about radiographic analyses, and the correlation between proper alignment restoration and long-term clinical outcome was unable to be assessed. Second, the operation was performed by experienced surgeons in our department, the results may differ although their operational skills are similar. Third, errors in the measurement of radiological parameters were possible due to the position and flexion contracture of patients when simple radiographs were taken.

In conclusion, the study showed that excessive lateral bowing of the femur has an important influence on the postoperative alignment with the IM femoral resection system. In this case, the application of the new EM system in TKA will optimize the alignment of lower extremities and the position of femoral components.

## Data Availability

The datasets will be available from the corresponding author on reasonable request.

## Abbreviations

IM: Intramedullary
TKA: Total knee arthroplasty
EM: Extramedullary
cFBA: Coronal femoral bowing angle
BMI: Body mass index
HKA: Hip-knee-angle
LDFA: Lateral distal femoral angle
MPTA: Medial proximal tibial angle
